# Circular RNA circHECTD1 prevents Diosbulbin-B-sensitivity via miR-137/PBX3 axis in gastric cancer

**DOI:** 10.1186/s12935-021-01957-1

**Published:** 2021-05-17

**Authors:** Yizhuo Lu, Long Li, Lianghui Li, Guoyang Wu, Guoyan Liu

**Affiliations:** 1 Department of General Surgery, Zhongshan Hospital, School of Medcine, Xiamen University, Xiamen, Fujian 361004 China; 2 Institute of Gastrointestinal Oncology, School of Medcine, Xiamen University, Xiamen, Fujian 361004 China; 3 Department of Gastrointestinal Surgery, Zhongshan Hospital, School of Medcine, Xiamen University, Room 203, 146 Hubin South Road, Siming District, Xiamen, Fujian 361004 China; 4 School of Pharmaceutical Sciences, Xiamen University, 361102 Xiamen, Fujian China

**Keywords:** circHECTD1, miR-137, PBX3, GC, DB-sensitivity

## Abstract

**Backgrounds:**

Gastric cancer (GC) is general disease in human digestive system with malignancy. Emerging findings indicated that hsa_circ_0031452 (circHECTD1) was strictly associated with carcinogenesis. Nevertheless, the role of circHECTD1 in drug-resistance still needed to be explained.

**Methods:**

Quantitative real-time polymerase chain reaction (qRT-PCR) was employed to examine the expression profiles of circHECTD1, microRNA (miR)-137, and pre-leukemia transcription factor 3 (PBX3). The function of circHECTD1 in tumorigenesis was evaluated via xenograft tumor model. The IC_50_ of Diosbulbin-B (DB) was detected using Cell Counting Kit-8 (CCK8). Cell-cycle and apoptosis were reckoned by flow cytometry. Besides, western blot was administrated to reckon the levels of PBX3 and cell apoptotic indicators. Moreover, the interrelation between miR-137 and circHECTD1 or PBX3 was expounded by dual-luciferase reporter, RNA immunoprecipitation (RIP) and RNA pull down assays.

**Results:**

We uncovered that circHECTD1 was ectopically up-regulated in GC tissues and cells. CircHECTD1 deficiency sensitized DB-treatment in DB-evoked AGS and HGC-27 cells. *In vivo* assay, circHECTD1 silencing led to the tumor reduction. Also, circHECTD1 served as miR-137 sponge in a sequence-complementary manner. Furthermore, transfection of miR-137 inhibitor markedly eliminated circHECTD1 absence-mediated promotion of DB-sensitivity in GC cells. Moreover, PBX3, a target of miR-137, play a DB-resistant role in GC cells. Fascinatingly, the deletion of PBX3 reversed the impact of miR-137 repression and circHECTD1 knockdown on DB-sensitivity *in vitro*.

**Conclusions:**

CircHECTD1 served as an oncogene by a novel miR-137/PBX3 axis, which might supply an underlying biomarker for the diagnosis and prognosis of GC management.

**Highlights**

1.  CircHECTD1 and PBX3 were overexpressed, whereas miR-137 was down-regulated in GC tissues and cells.
2.  The absence of circHECTD1 caused the curb of tumor growth *in vivo*.
3.  The depletion of circHECTD1 expedited DB-sensitivity via miR-137/PBX3 axis *in vitro*.

## Backgrounds

Gastric cancer (GC) is a frequent malignant tumor in human digestive section, and surgical resection is recently deemed as the only essential therapy for GC in the clinic [1]. However, the occurrence of aggressive metastasis seriously restricts the efficiency of surgery [2]. *Dioscorea bulbifera L*, a Chinese medicine, has been suggested to be related to cancer treatment [3, 4], and Diosbulbin-B (DB) is the master compound of the Chinese herb [5]. However, a high dose of DB can result in hepatotoxicity, thereby limiting its application for GC therapeutic strategy [6–8]. Understanding the potential knowledge of the DB-dose and enhancing the sensitivity of DB might solve this barrier.

Circular RNAs (circRNAs), the novel endogenous non-coding RNAs (ncRNAs), are strictly implicated in the pathogenesis of human tumors, including GC [9–11]. For circRNAs, it unlike the well-known long non-coding RNAs (lncRNAs), have the closed-loop with the 3’ and 5’ ends joined together. In addition, thousands of existed circRNAs have been identified recently [12]. For example, the low aberrant expression of has_circ_0000096 is closely involved in cell growth and metastasis in GC cells [13]. Besides, earlier works also focus on the regulatory function of circRNAs in drug-resistance in human cancers. Zhou *et al*. document that hsa_circ_0004015 can mediate aggressive phenotypes and drug-resistance by sponging miR-1183 in non-small cell lung cancer [14]. Has_circ_0000199 can enhance GC resistance to cisplatin via inactivating miR-198 in GC [15]. Interestingly, hsa_circ_0031452 (circHECTD1) acts as an oncogenic mediator to modulate the progression of GC through glutaminolysis by sponging microRNA (miR)-1256 [16]. Nevertheless, the relationship between circHECTD1 and drug-resistance still under-reported. Currently, we aimed to explain the regulatory mechanism of circHECTD1 in altering DB-resistance in GC.

Up to date, the role of miRNAs attracts more attention, and they are sponged by circRNAs or long non-coding RNAs (lncRNAs) in competing endogenous RNAs (ceRNAs) manner [17, 18]. Emerging evidence reveals that miRNAs participate in the pathogenesis of GC. Especially, miR-7-5p works as a tumor suppressor in several cancers [19, 20], including GC [21]. After genetic screen identification, miR-372 acts as an oncogene in testicular germ cell tumors [22]. MiR-137 was generally low-expressed in GC [23]. Accordingly, miRNAs are uncovered in diverse organisms and serve as the modulator of the level of the target gene via complex processes [24]. For example, miR-137 exerts its inhibitory effect on carcinogenesis by targeting cyclooxygenase-2 in GC [25]. Notably, DB is a regulator for the level of miRNAs [8]. Understanding the regulatory function between DB-resistance and miR-137 is available to search for the drug-resistance for the therapy of human cancers. Pre-leukemia transcription factor 3 (PBX3) is the main mediator in the onset and progression of aggressive phenotypes in human cancers [26]. We hypothesized that PBX3 might be connected with miR-137 in GC.

In this report, we determined the expression patterns of circHECTD1, miR-137, and PBX3 in GC tissues and cell lines, the partial work pathway of them in mediating DB-resistance was the novelty of this paper.

## Materials and methods

### Clinical specimens

Paired GC tissues (n = 25) and adjacent normal tissues (n = 25) were donated by the GC patients who underwent diagnosis and therapy at Zhongshan Hospital, School of Medcine, Xiamen University. The clinical trial was conducted in accordance with the ethical principles of the Declaration of Helsinki (as revised in 1983), the International Conference on Harmonization of Good Clinical Practice (ICH-GCP, 1996), and local laws. All the enrolled donators did not receive preoperative treatment, including radiotherapy or chemotherapy. The eligible tissues were immediately maintained in liquid nitrogen. The clinicopathologic features of these patients were presented in Table 1. Written informed consents were acquired from all patients prior to surgery, and this research was operated according to the approval of the Ethics Committee of the Zhongshan Hospital, School of Medcine, Xiamen University.

**Table 1:** The relationship between the expression of circHECTD1 and clinicopathological parameters in GC patients

Clinicopathological factors	Number	circHECTD1 expression		P value
		Down	High	
Age				
< 50 years	13	5	8	> 0.05
≥ 50 years	12	6	6	
Gender				
Female	12	5	7	> 0.05
Male	13	6	7	
Tumor size				
> 3 cm	16	6	10	< 0.05
≤ 3 cm	9	4	5	
Lymph node metastasis				
Negative	15	7	8	< 0.05
Positive	10	4	6	
TNM stage				
I–II	17	7	10	< 0.05
III–IV	8	4	4	

### Cell culture

The human cell lines (AGS and HGC-27) and normal human gastric mucosal epithelial cell line GES-1 were purchased from Shanghai Institute of Cell Biology, Chinese Academy of Sciences (Shanghai, China). Roswell Park Memorial Institute-1640 (RPMI-1640; Hyclone, Logan, UT, USA) with supplement reagents, including 10 % fetal bovine serum (FBS; Gibco, Carlsbad, CA, USA) and 1⋅antibiotics (Hyclone, 100 U/mL penicillin and 100 mg/mL streptomycin) were applied for cell culture in a humidified atmosphere (5 % CO_2_) at 37℃. Except that, DB (APExBIO Technology, Austin, TX, USA) with different doses (0, 3.125, 6.25, 12.5, 25, 50, or 100 µM) served as the inductor to stimulate GC cells.

### Cell transfection

Small interfering RNA (siRNA) targeting circHECTD1 (si-circHECTD1) and PBX3 (si-PBX3), as well as control of siRNA (si-NC), were constructed in Genewiz (Suzhou, China). Oligonucleotides, including miR-137 mimic (miR-137), inhibitor (anti-miR-137), and their relative controls (miR-NC and anti-miR-NC) were purchased from GenePharma (Shanghai, China). Transient transfection was implemented by means of Lipofectamine 3000 (Invitrogen, Carlsbad, CA, USA) on the base of the user’s guidebook. Besides, the PBX3 full length cDNA was inserted into the pcDNA3.1 vector (Invitrogen) to generate the PBX3 overexpression vector, and empty pcDNA3.1 vector was used as the control. Furthermore, short hairpin (shRNA) negative circHECTD1 (sh-HECTD1) and its scramble (sh-NC) was designed in GenePharma and used for establishing stably transfected cells via lentivirus mediation. The sequences of siRNA, shRNA and oligonucleotides used in this research were shown in Table 2.

**Table 2:** Sequences of siRNA, shRNA and oligonucleotides used in this research

Gene	siRNA Sequence (5’-3’)
si-circHECTD1	CGGTTGTACGCAAGGTTGATC
si-NC	
Sense	GAUUGUUCUGAGAGACGAAGA
Antisense	UUCGUCUCUCAGAACAAUCUG
si-PBX3	
Sense	UUCUUCGAACGUGUCACGUTT
Antisense	ACGUGACACGUUCGGAGAATT
anti-miR-137	CUACGCGUAUUCUUAAGCAAUAA
anti-miR-NC	GGUUCGUACGUACACUGUUCA
miR-137	UUAUUGCUUAAGAAUACGCGUAG
miR-NC	GGUUCGUACGUACACUGUUCA
sh-circHECTD1	
Sense	GATCCGCACGGTTGTACGCAAGGTTGTTCAAGAGACAACCTTGCGTACAACCGTGTTTTTTGGAAG
Antisense	AATTCTTCCAAAAAACACGGTTGTACGCAAGGTTGTCTCTTGAACAACCTTGCGTACAACCGTGCG
sh-NC	
Sense	CACCGTTCTCCGAACGTGTCACGTCAAGAGATTACGTGACACGTTCGGAGAATTTTTTG
Antisense	GATCCAAAAAATTCTCCGAACGTGTCACGTAATCTCTTGACGTGACACGTTCGGAGAAC

### Quantitative real‐time polymerase chain reaction (qRT-PCR) assay

Total RNA from tissues and cultured cells was purified and extracted by Trizol reagent (Invitrogen) following the producer’s manuals. The harvested RNA was reversely transcribed into complementary DNA (cDNA) with the use of the TaqMan Reverse Transcription Reagents (Applied Biosystems, Foster City, CA, USA). The synthesized cDNA was measured through the SYBR Green PCR Master Mix (Applied Biosystems) via the 2^−ΔΔCt^ method. The relative quantity was calculated after normalization by glyceraldehyde-3-phosphate dehydrogenase (GAPDH; for circHECTD1 and PBX3) and U6 (for miR-137). The primers used in this study were shown: circHECTD1 (Forward: 5’-GTGGACTGGCTAACCTGCAT-3’, Reverse: 5’- TCCTGTGGAAATGGTGCTGT-3’); miR-137 (Forward: 5’-TTATTGCTTAAGAATACG-3’, Reverse: 5’-AACTCCAGCAGGACCATGTGAT-3’); PBX3 (Forward: 5’-GAGCTGGCCAAGAAATGCAG-3’, Reverse: 5’-GGGCGAATTGGTCTGGTTG-3’); GAPDH (Forward: 5’-ACTCCTCCACCTTTGACGC-3’, Reverse: 5’-GCTGTAGCCAAATTCGTTGTC-3’). U6 (Forward: 5’-CTCGCTTCGGCAGCACA-3’, Reverse: 5’-AACGCTTCACGAATTTGCGT-3’).

### Xenograft tumor model

BALB/c nude mice were purchased from Vital River Laboratory Animal Technology (Beijing, China) and the animal feeding and experimental procedures were ratified by the Institutional Animal Care and Use Committee of Zhongshan Hospital, School of Medcine, Xiamen University. Briefly, the nude mice (n = 6/group, 5-week-old) were divided into two groups randomly. And stably expressed AGS cells (5 × 10^6^ cells) infected with sh-circHECTD1 or sh-NC plasmid DNA were injected into the left flank of the dorsum subcutaneously. The tumor size was measured and recorded per week, and the tumor volume was calculated: volume = length⋅width^2^⋅0.5. After injection for 4 weeks, all the experimental mice were sacrificed and tumor weight was tested.

### Cell counting Kit-8 (CCK8)

The GC cells (AGS and HGC-27) and GES-1 cells were treated accordingly. Next, the cells were transfected vectors when the confluence reached about 70 %. After incubation with DB for 48 h, cell viability was determined by means of CCK8. Briefly, the CCK8 reagent (10 µL/well) was supplemented into cellular wells and incubated for another 2 h. Ultimately, the value of the optical density (OD) was evaluated at 450 nm to quantify the cell viability. Besides, the concentration of DB represented the half-maximal inhibitory concentration (IC_50_) when 50 % of the cells were dead.

### Flow cytometry assay

For the cell-cycle assay, cells were collected at 48 h post-transfection, and 70 % ethanol was employed to fix the above cells overnight at 4 ℃. After that, fixed cells were stained for half an hour at room temperature after Propidium iodide (PI; Beyotime, Shanghai, China) addition. Subsequently, cell-cycle was examined by flow cytometry using the FACS Calibur system (BD Biosciences, San Jose, CA, USA), and the data were analyzed with the help of ModFit 3.0 software (BD Biosciences). For cell apoptosis assay, the AGS and HGC-27 cells were incubated for 48 h with DB treatment and vectors transfection. After that, the tested cells were harvested with Phosphate Buffer solution (PBS) and double-stained by Annexin V/PI kit (SenBeiJia Biological Technology Co., Ltd., Nanjing, China) following user’s guidebook. After incubation for 20 min without light, the apoptotic signals were captured by flow cytometry (BD Biosciences).

### Western blot assay

As previously described [27], the protein lysed from tissues and cells were separated on the sodium dodecyl sulfate-polyacrylamide gel and then blotted onto the polyvinylidene fluoride membranes (PVDF; Millipore, Bedford, MA, USA). Then, the isolated proteins were incubation with special primary antibodies (Abcam, Cambridge, MA, USA), including anti-PBX3 (1:6000, ab109173), anti-B-cell lymphoma-2 (Bcl-2, 1:1000, ab32124), anti-Bcl-2-Associated X (Bax, 1:7000, ab32503), and anti-β-actin (1:6000, ab8226). After incubation overnight at 4 ℃, the corresponding secondary antibodies were adopted to combine and amplify the protein signals. Also, the proteins on the membranes were visualized and quantified after addition with the reagent combination of an enhanced chemiluminescence detection kit (Millipore).

### Dual‐luciferase reporter assay

The sequences of miR-137 containing the wild type or mutant regions of circHECTD1 were amplified and cloned into the pmirGLO vectors (Promega, Madison, WI, USA), and named as WT-circHECTD1 and MUT-circHECTD1. Similarly, the binding sites of miR-137 combined with the 3’UTR of PBX3 (wild type and mutant) were also used to construct WT-PBX3 and MUT-PBX3 reporters. The above reporters were co-transfected with miR-137 or miR-NC into AGS and HGC-27 cells, respectively. Then, the treated cells were harvested, and the luciferase activity in the lysates was assessed and recorded by using the Dual-luciferase reporter gene system (Promega). All the assays were run in triplicate.

### RNA immunoprecipitation (RIP) assay

RIP assay was conducted with the help of Magna RIP RNA-Binding Protein Immunoprecipitation Kit (Millipore) as per the protocols. Briefly, RIP lysis buffer supplemented protease and RNase inhibitors (Millipore) was used for cell lysis. And the lysates were incubated with anti-Argonaute2 (Ago2; Millipore) or anti-immunoglobulin G (IgG; Millipore) overnight at 4 ℃. The co-precipitated RNAs were eluted with RIP buffer containing proteinase K. The enrichment of circHECTD1 or PBX3 was detected through adopting qRT-PCR assay.

### RNA pull‐down

RNA pull-down assay was conducted by using the Pierce Magnetic RNA–Protein Pull down Kit (Thermo Fisher Scientific). In brief, the biotin-labeled miR-NC probe and miR-137 probe were obtained from Sangon (Shanghai, China). The lysate samples of AGS and HGC-27 cells were incubated with miR-NC probe and miR-137 probe and streptavidin magnetic beads at 4 °C overnight. After washing with the wash buffer, the RNA complexes were purified and the level of circHECTD1 was measured by qRT-PCR.

### Statistical analysis

The data processed by GraphPad Prism 7 software were expressed as mean ± Standard Deviation (SD). A Student’s *t*-test was recruited for the comparison between two groups, and one-way analysis of variance with Tukey test was administrated to analyze the difference among multiple groups. *P* < 0.05 was the statistical indicator for the significant difference.

## Results

### CircHECTD1 and PBX3 were upregulated while miR-137 was decreased in GC tissues and cells

**Fig. 1:**
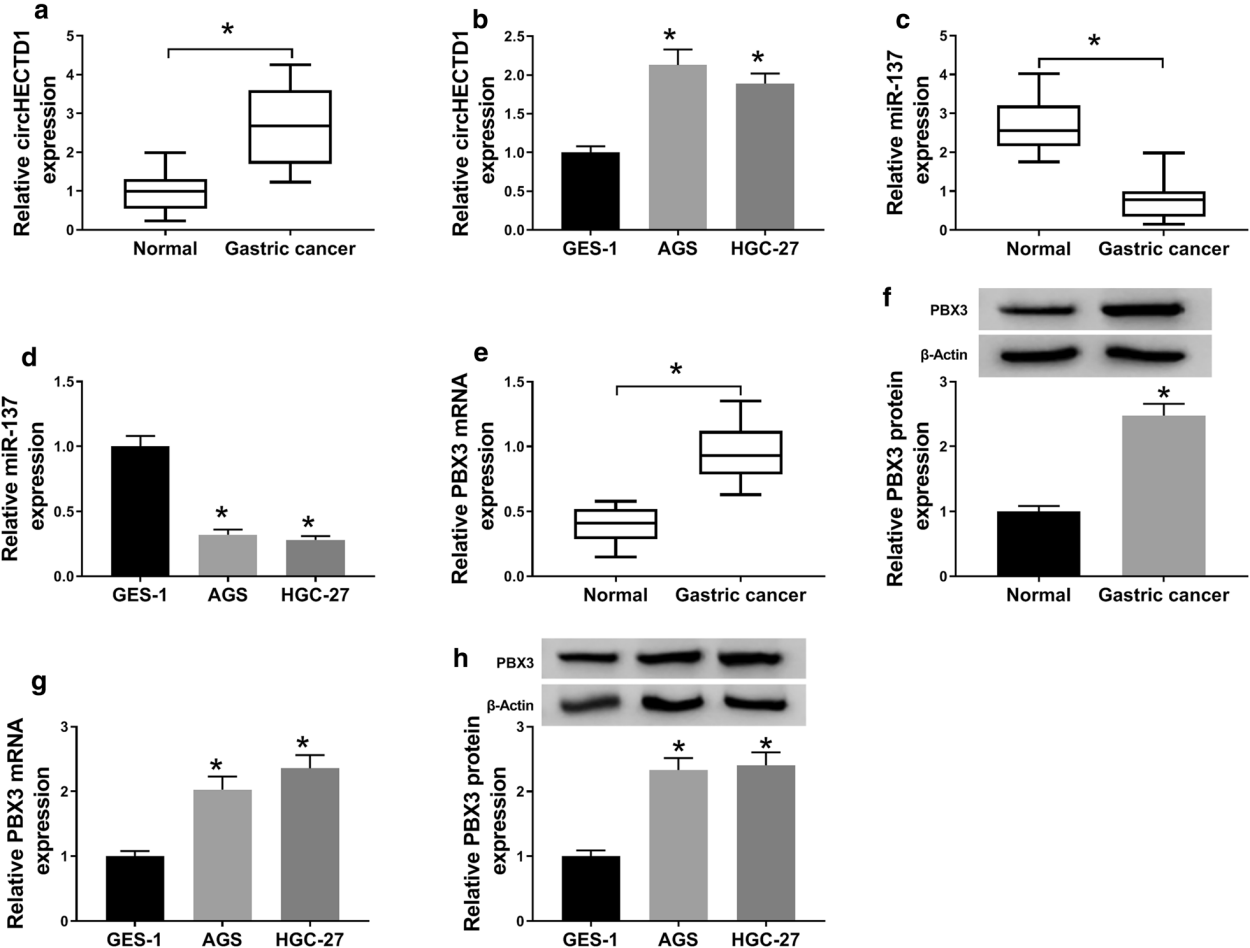
The levels of circHECTD1, miR-137 and PBX3 in GC tissues and cells. **a** Relative level of CircHECTD1 in 25 pairs of GC tissues and adjacent normal tissues (n = 25) was estimated by qRT-PCR. **b** Relative level of CircHECTD1 in GC cells (HGC-27 and AGS) and the normal human gastric mucosal epithelial cells GES-1. **c**, **d** The level of miR-137 in GC tissues (n = 25) and cells (HGC-27 and AGS), as against with normal tissues (n = 25) and cells (GES-1). **e**–**h** The mRNA and protein levels of PBX3 in GC tissues (n = 25) and cells (HGC-27 and AGS), as well as in paired normal tissues (n = 25) and cells (GES-1), were analyzed by qRT-PCR and western blot. The data represent the means ± SD of three different experiments. *P < 0.05

To further explore the biological function of circHECTD1, miR-137 and PBX3 in GC, qRT-PCR assay was on 25 cases of GC tissues and paired adjacent normal tissues (n = 25). As shown in Fig. 1a, circHECTD1 was obviously up-regulated in GC tissues in contrast with adjacent normal tissues. And a similar expression tendency of circHECTD1 was observed in AGS and HGC-27 cells compared with GES-1 cells (Fig. 1b). Conversely, miR-137 expression in GC tissues (n = 25) and cells was significantly decreased compared with the adjacent normal tissues (n = 25) and normal cells (GES-1) (Fig. 1c, d). Besides, further investigation indicated that PBX3 mRNA and protein levels in GC tissues (n = 25) and cells were significantly higher than that in adjacent normal tissues (n = 25) and cells (Fig. 1 e, h).

### CircHECTD1 silencing curbed GC tumor growth in vivo

**Fig. 2:**
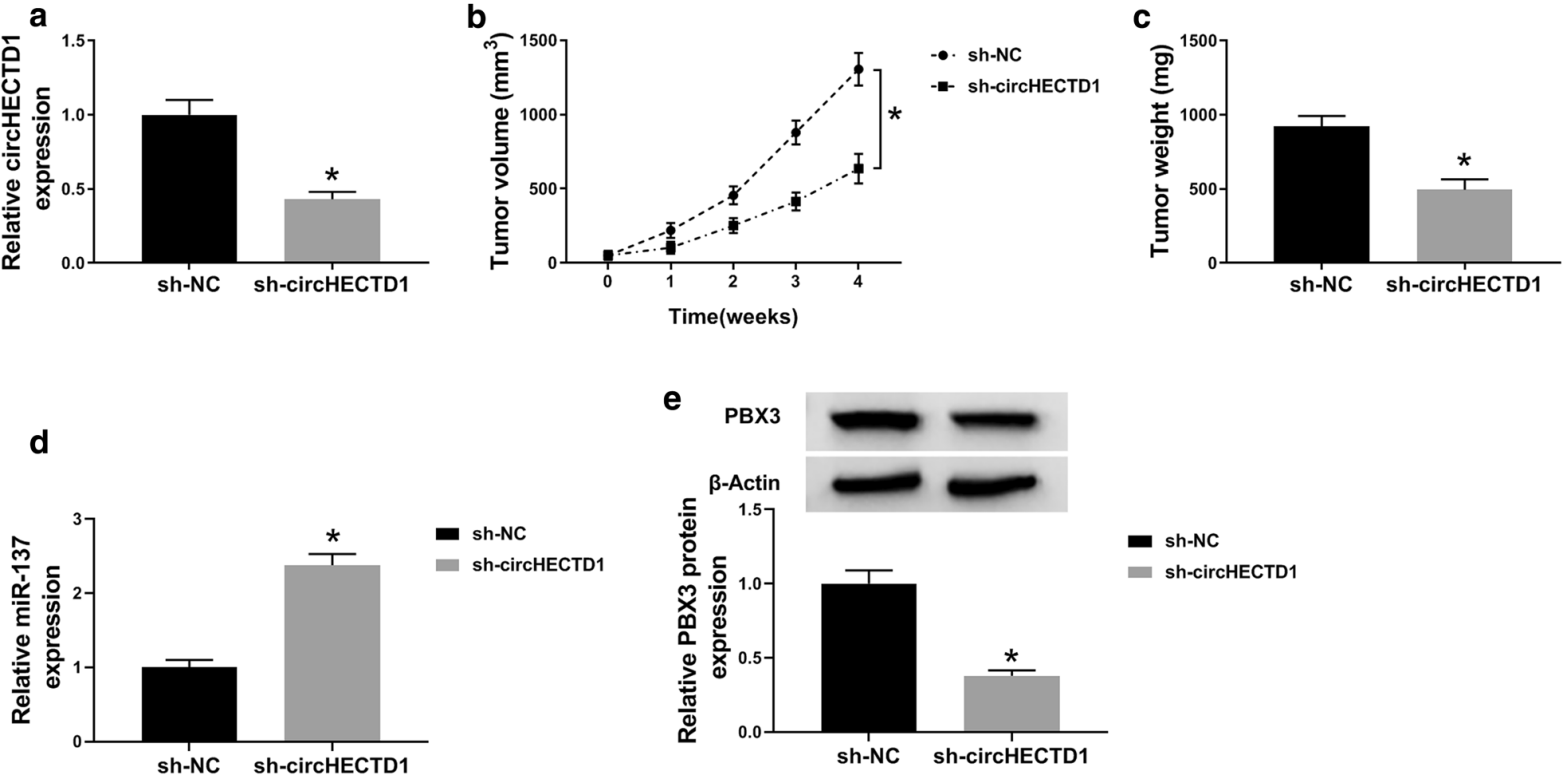
CircHECTD1 knockdown suppressed GC tumor growth in vivo. Nude mice were injected with lentivirus-mediated AGS cells with sh-circHECTD1 or sh-NC treatment. **a** QRT-PCR analysis for the expression of circHECTD1 in the two groups. **b**, **c** The data analysis for the tumor volume and weight in excised tissues. **d** QRT-PCR analysis for the level of miR-137 in the two groups. **e** Western blot analysis for the relative protein level of PBX3 in the two groups. The data represent the means ± SD of three different experiments. *P < 0.05

As circHECTD1 functions as a tumor suppressor in GC [16], we further investigated the effect of circHECTD1 silencing on tumor growth *in vivo*. Firstly, stably expressed AGS cells (transfected with sh-circHECTD1 or sh-NC) were injected into the flank of mice, and the level of circHECTD1 diminished in sh-circHECTD1-mediated group (Fig. 2a). As expected, the significant decrease of circHECTD1 caused the curb of tumor growth, as the repression of tumor volume and weight in xenograft tissues (Fig. 2b, c). Furthermore, qRT-PCR and western blot respectively revealed the enhancement of miR-137 expression and the reduction of PBX3 protein level in mice with circHECTD1 silence *in vivo* (Fig. 2d, e). These results revealed that circHECTD1 was a cancer-associated circRNA.

### CircHECTD1 absence reinforced DB-sensitivity in GC cells

**Fig. 3:**
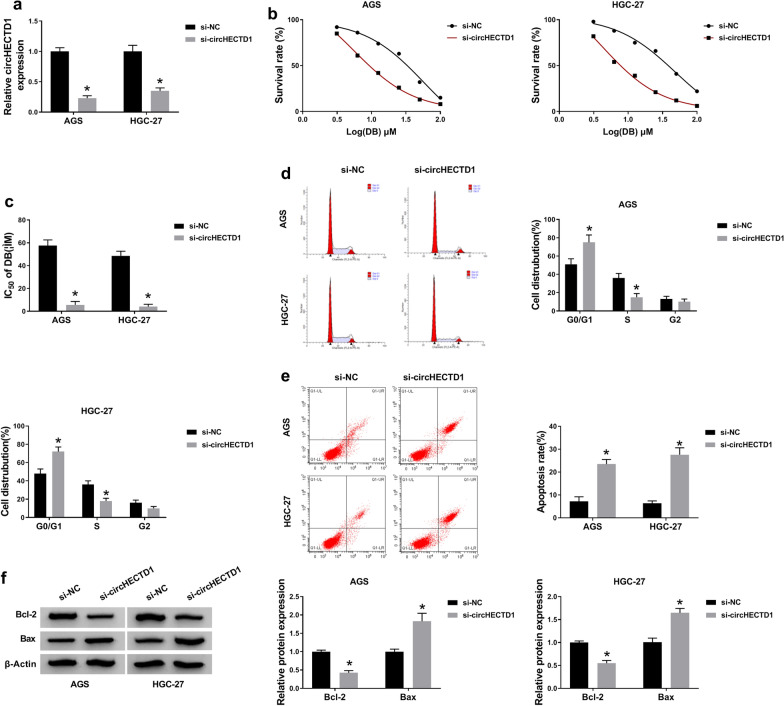
CircHECTD1 absence could reinforce DB-sensitivity in GC cells. AGS and HGC-27 cells were transfected with si-circHECTD1 or si-NC, severally. **a** Knockdown efficiency of si-circHECTD1 transfection in altering the level of circHECTD1 in the two GC cells. **b**, **c** MTT analysis for the IC_50_ of DB in treated cells. **d** The effect of circ-HECTD1 silencing on cell-cycle. **e** Flow cytometry analysis for the apoptosis of the two GC cells. **f** Relative levels of apoptosis-related proteins (Bcl-2 and Bax) in circHECTD2 deficient AGS and HGC-27 cells. The data represent the means ± SD of three independent assays. *P < 0.05

Based on the ectopic expression of circHECTD1, we conjectured that it might participate in drug-resistance in GC. To illustrate our guess, si-circHECTD1 or si-NC was transfected into AGS and HGC-27 cells. Results from qRT-PCR exhibited that circHECTD1 was clearly declined in si-circHECTD1-mediated GC cells (Fig. 3a). Subsequently, AGS and HGC-27 cells were treated with different doses of DB, the IC_50_ was sharply decreased in circHECTD1-silenced GC cells (Fig. 3b, c). And the dose of DB resulted in the 50 % percent death of cells was selected for the subsequent experiments. As depicted in Fig. 3d, cell-cycle was particularly hindered after circHECTD1 absence resulting in elevated cells percentage in the G0/G1 phase in DB-induced AGS and HGC-27 cells. In addition, an effective promotion of cell apoptosis was observed in DB-exposed GC cells with circHECTD1 deficiency (Fig. 3e). Furthermore, the low level of Bcl-2 and high expression of Bax in si-circHECTD1-transfected GC cells also elucidated the above conclusion about cell apoptosis (Fig. 3f). All the findings determined that circHECT1 deletion could expedite the DB-sensitivity *in vitro*.

### CircHECTD1 was a sponge of miR-137

**Fig. 4:**
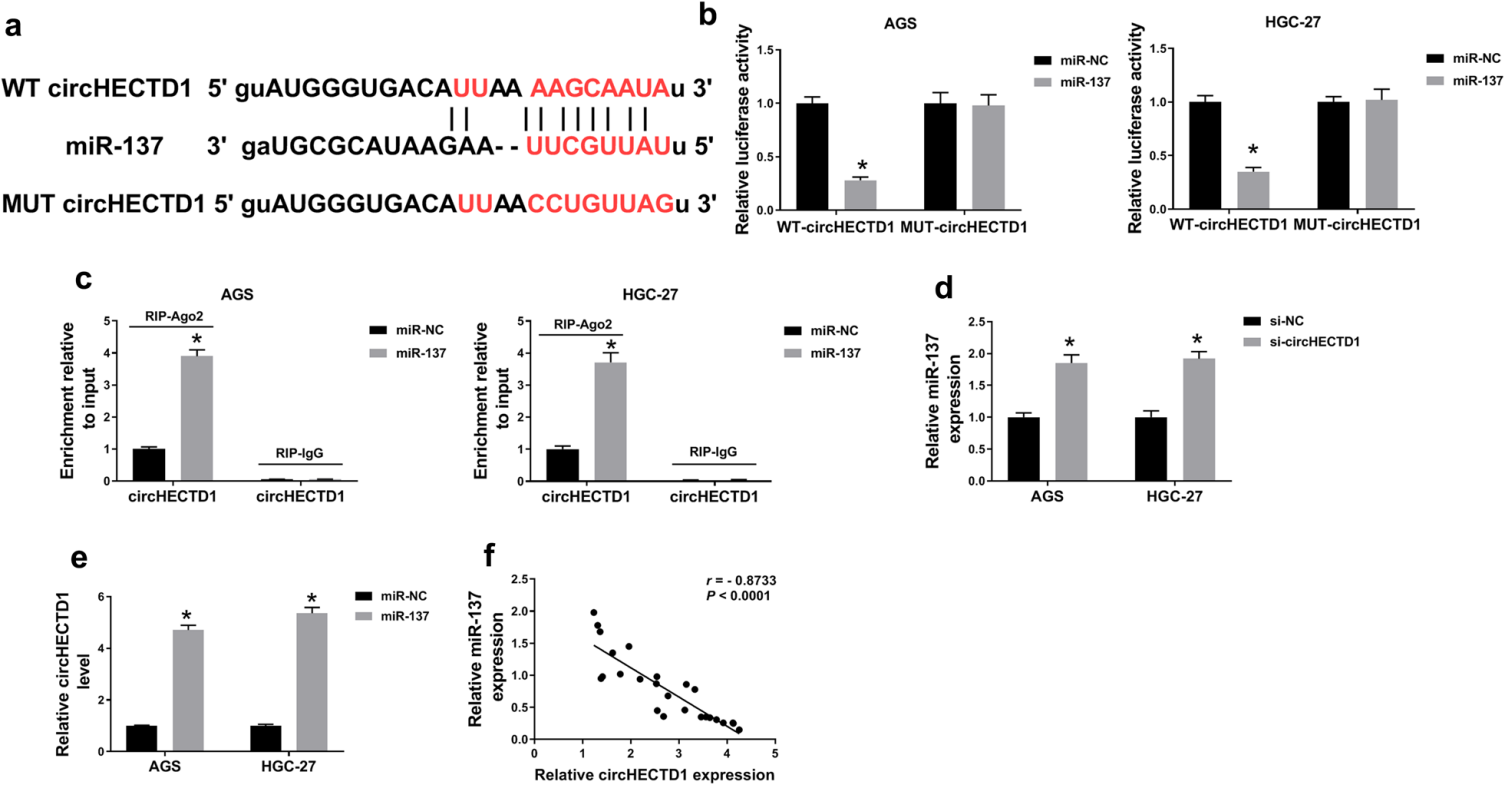
CircHECTD1 acted as a sponge for miR-137. **a** The complementary binding sites between circHECTD1 and miR-137 were predicted by StarbaseV3.0. **b**, **c** Dual-luciferase reporter for the interaction between miR-137 and circHECTD1. **d** Relative level of miR-137 in circHECTD1-silenced AGS and HGC-27 cells. **e** RNA pull down assay for the enrichment of circHECTD1 in cells with miR-NC probe or miR-137 probe incubated. **f** Correlation between miR-137 and circHECTD1 in 25 cases of GC tissues was estimated. The data of the three different tests were shown as means ± SD. *P < 0.05

In view of the functional verification of circHECTD1, the targets of circHECTD1 needed to be sought. As predicted by starBase, the binding sites between circHECTD1 and miR-137 were found and shown in Fig. 4a. Dual-luciferase reporter assay uncovered that miR-137 constrained the luciferase activity of WT-circHECTD1 group, not the MUT-circHECTD1 group (Fig. 4b). Besides, RIP assay indicated that circHECTD1 was enriched in Ago2 incubated group, not IgG group (Fig. 4c), and transfection of miR-137 mimic further increased the interaction between circHECTD1 and Ago2, revealing the existence of miR-137 and circHECTD1 in a RNA-induced silencing complex (RISC). Furthermore, miR-137 level was increased in DB-evoked AGS and HGC-27 cells with circHECTD1 knockdown (Fig. 4d). In addition, RNA pull-down assay was conducted to verify this interaction. As displayed in Fig. 4e, circHECTD1 was pulled down by miR-137 probe, but no such effect was found in cells with miR-NC probe incubation. Moreover, we found that miR-137 was inversely correlated (*r* = − 0.8733, *P* < 0.0001) with circHECTD1 in 25 cases of GC tissues (Fig. 4f). The above results demonstrated that circHECTD1 negatively controlled the levels of miR-137 in GC via sponging miR-137.

### **Circ-HECTD1 suppressed DB-sensitivity via sponging miR-137 in AGS and HGC-27 cells**

**Fig. 5:**
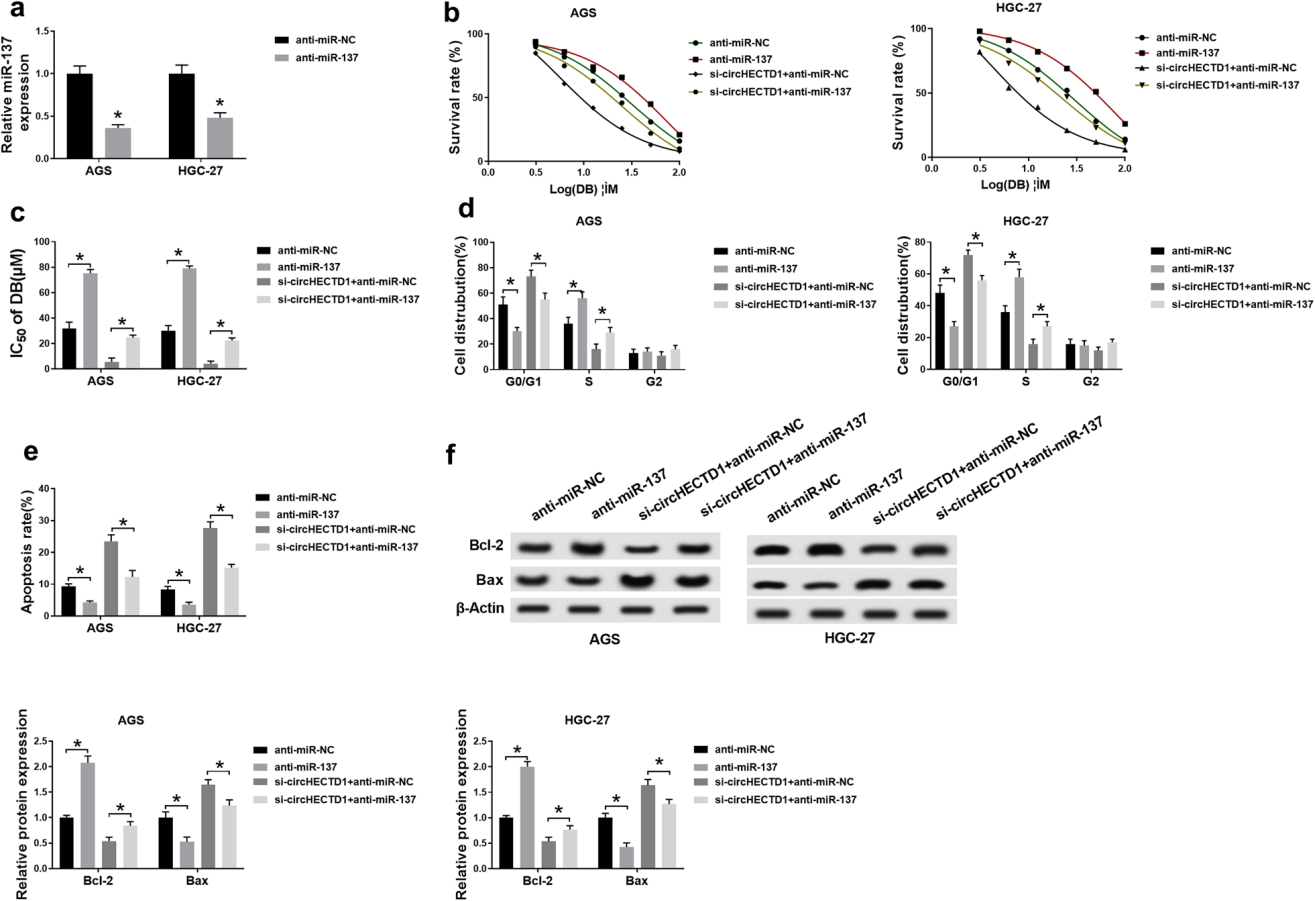
Circ-HECTD1 functioned its repressive effect on DB-sensitivity via miR-137 in AGS and HGC-27 cells. **a** QRT-PCR analysis for the level of miR-137 in AGS and HGC-27 cells with anti-miR-137 or anti-miR-NC transfection. **b**–**f** AGS and HGC-27 cells with DB induction were introduced with anti-miR-NC, anti-miR-137, si-circHECTD1 + anti-miR-NC, or si-circHECTD1 + anti-miR-137, respectively. **b,** **c** The IC_50_ of DB in the two GC cells. **d** The influence of si-circHECTD1 or anti-miR-137 on cell cycle distribution. **e** The apoptotic ratio in si-circHECTD1 or anti-miR-137-mediated AGS and HGC-27 cells. **f** Relative levels of Bcl-2 and Bax in GC cells. The data represent the means ± SD of three independent experiments. *P < 0.05

Given the above descriptions, rescue assays were carried out to clarify the functional mechanism between circHECTD1 and miR-137. Firstly, we corroborated that anti-miR-137 transfection indeed restrained the level of miR-137 in AGS and HGC-27 cells (Fig. 5a). Next, anti-miR-137 alone or along with si-circHECTD1 was introduced into the two GC cells with DB treatment. MTT analysis displayed that miR-137 inhibition led to the increase of the IC_50_ of DB, and could reverse circHECTD1 silencing-mediated inhibitory effect on the IC_50_ of DB in DB-stimulated AGS and HGC-27 cells (Fig. 5b, c). Synchronously, miR-137 absence suppressed cell-cycle arrest in DB-exposed GC cells, the acceleratory influence of circHECTD1 repression on cell-cycle arrest was overturned after miR-137 inhibition (Fig. 5d). Moreover, cell apoptosis was obviously hampered as a result of miR-137 reduction. Interestingly, the promoting impact of si-circHECTD1 supplement on cell apoptosis was partially abrogated after simultaneous retard of miR-137 in AGS and HGC-27 cells with DB induction (Fig. 5e, f). Namely, circHECTD1 was a sponge of miR-137 to modify DB-sensitivity in GC cells stimulated by DB.

### PBX3 was directly targeted by miR-137

**Fig. 6:**
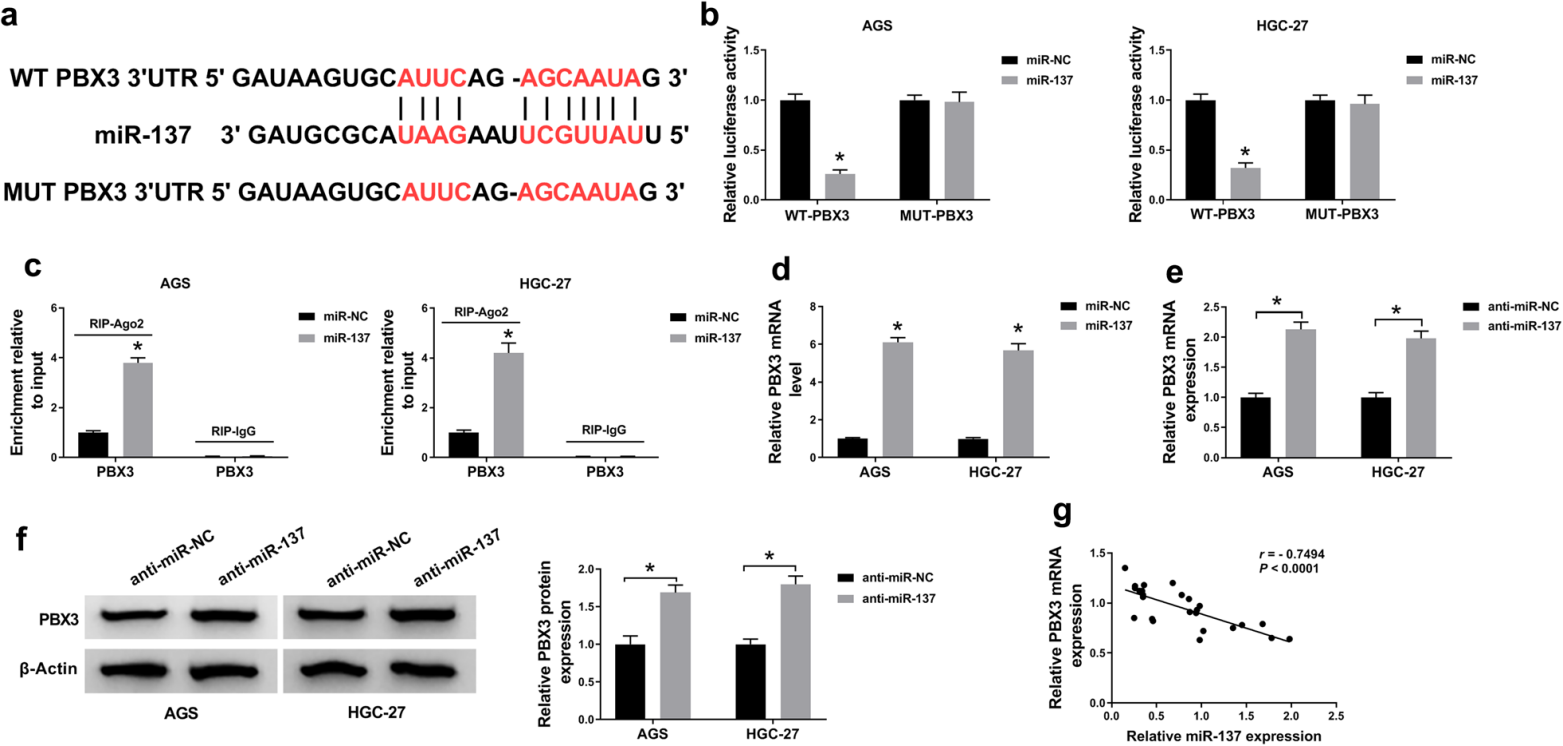
PBX3 was directly targeted by miR-137. **a** Complementary binding sites between miR-137 and PBX3 were predicted by Targetscan software. **b** The luciferase activities of AGS and HGC-27 cells with WT-PBX3 or MUT-PBX3 and miR-137 or miR-NC co-transfection. **c** RIP analysis for the validation of the relationship between miR-137 and PBX3. **d** RNA pull down assay for PBX3 level in cells with miR-NC probe or miR-137 probe incubated. **e**,  **f** The impact of miR-137 depletion on the level of PBX3 in GC cells. **g** The correlation between miR-137 and PBX3 in 25 cases of GC specimens. The data from the three different assays were presented as means ± SD. *P < 0.05

Considering that miR-137 was tightly related to DB-resistance, the next purpose of us was to search for the possible targets of miR-137. As shown in Fig. 6a, prediction from Targetscan software unraveled that PBX3 was a candidate gene of miR-137. And the relationship between them was validated via dual-luciferase reporter and RIP assays. Specifically, miR-137 reduced the luciferase activity of WT-PBX3 reporter, while had no conspicuous effect on luciferase activity of MUT-PBX3 reporter in GC cells (Fig. 6b). Also, the abundance of PBX3 was apparently higher in the miR-137-transfected group (Fig. 6c). Moreover, RNA pull down assay uncovered that PBX3 was only pulled down by miR-137 probe (Fig. 6d). Meanwhile, downregulation of miR-137 elevated the mRNA and protein levels of PBX3 in GC cells (Fig. 6e, f). Moreover, PBX3 was negatively correlated (*r* = −0.7494, *P* < 0.0001) with miR-137 level in the clinical GC subjects (Fig. 6g). In brief, miR-137 directly targeted PBX3 in GC.

### **MiR-137 interacted with PBX3 to regulate DB-sensitivity of GC cells**

**Fig. 7:**
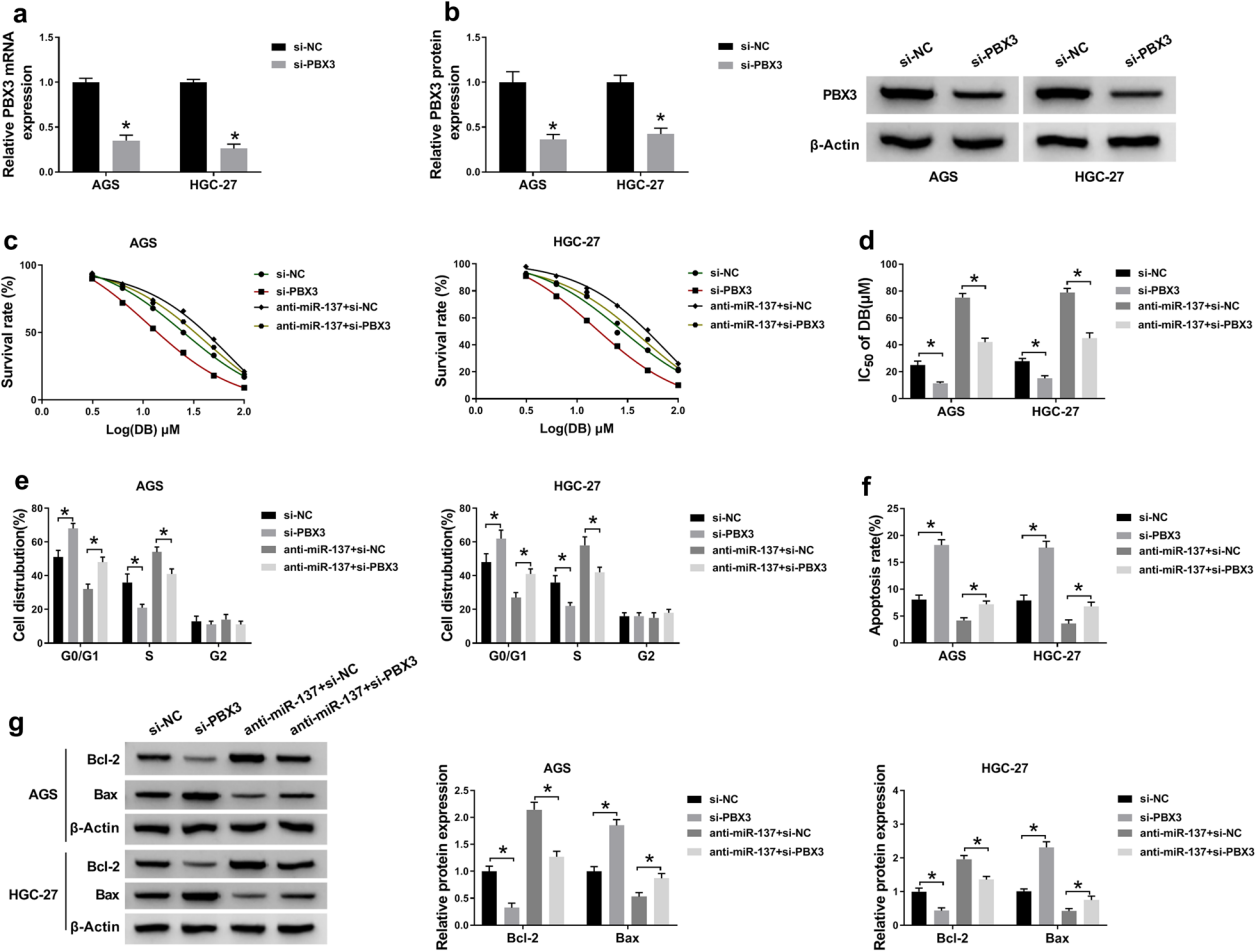
MiR-137 modulated DB-resistance by targeting PBX3 in the two GC cells. **a**, **b** The knockdown efficiency of si-PBX3 transfection in PBX3 level. **c**–**g** si-NC, si-PBX3, anti-miR-137 + si-NC, or anti-miR-137 + si-PBX3 was transfected into AGS and HGC-27 cells with DB exposure. **c**, **d** MTT analysis for the IC_50_ values (µM) of DB in the two GC cells. **e**, **f** Flow cytometry analysis for **e** cell-cycle and **f** cell apoptosis *in vitro*. **g** Relative levels of Bcl-2 and Bax in treated AGS and HGC-27 cells. The data of the three different tests were exhibited as means ± SD. *P < 0.05

On the basis of the interrelation between miR-137 and PBX3, we attempted to shed light on the functional regulation between them. Firstly, after transfection with si-PBX3, the level of PBX3 was sharply weakened in AGS and HGC-27 cells (Fig. 7a, b). And the functional mechanism was expounded via rescue assay. As grafted in Fig. 7c, d, PBX3 depletion not only retarded the IC_50_ of DB, but also restored miR-137 inhibition-mediated increase of DB-IC_50_ in DB-stimulated AGS and HGC-27 cells. Then, we also clarified that PBX3 absence contributed to cell-cycle arrest. Also, the influence of miR-137 deletion on cell-cycle arrest was overturned after regain of si-PBX3 in the two GC cells with DB treatment (Fig. 7e). Not only that, cell apoptosis was remarkably augmented when PBX3 was diminished, and reintroduction of si-PBX3 greatly abolished the repressive impact of miR-137 inhibition on cell apoptosis in DB-evoked AGS and HGC-27 cells (Fig. 7f, g). Thus, miR-137 suppressed the expression of PBX3 to enhance the sensitivity of GC cells to DB treatment.

### **CircHECTD1 regulated DB-sensitivity of GC cells by sponging miR-137 to elevate the expression of PBX3**

**Fig. 8:**
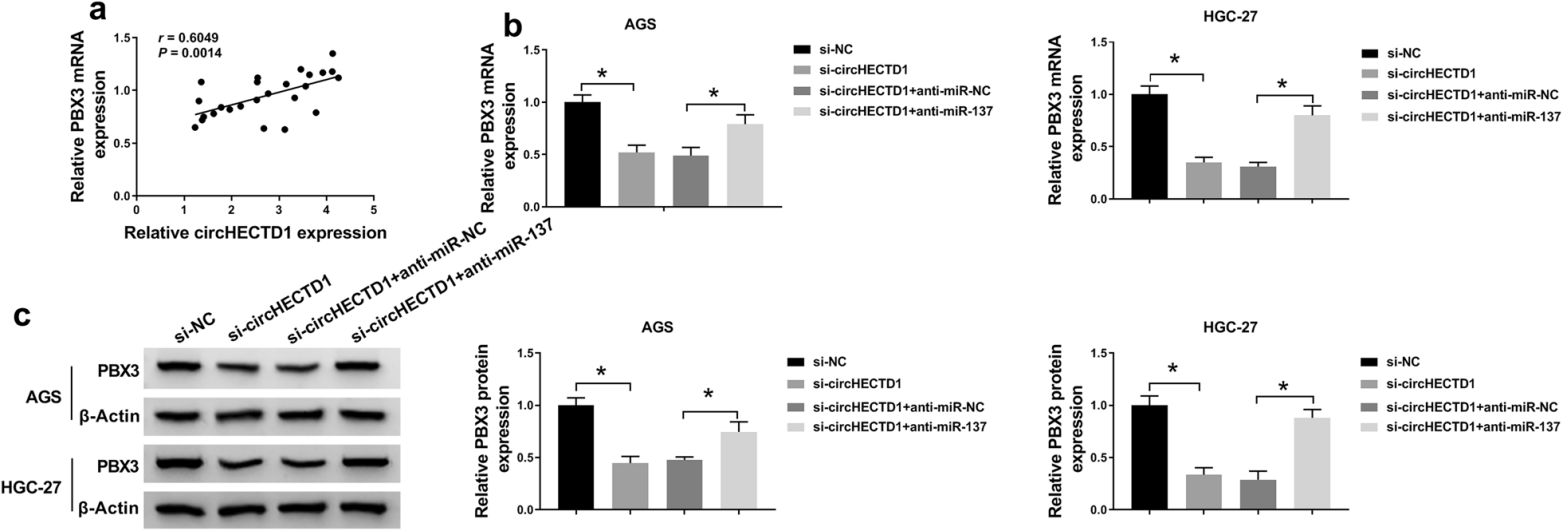
CircHECTD1 upregulated PBX3 expression by sponging miR-137 in GC. **a** Correlation between circHECTD1 and PBX3 expression levels in 25 cases of GC tissues was analyzed by Pearson’s correlation analysis. **b**, **c** Si-NC, si-circHECTD1, si-circHECTD1 + anti-miR-NC, or si-circHECTD1 + anti-miR-137 was introduced into AGS and HGC-27 cells with DB stimulation, respectively. QRT-PCR and western blot analyses for the level of PBX3 in transfected cells at the aspects of mRNA and protein expression. All the data from three different experiments were shown as means ± SD. *P < 0.05

**Fig. 9:**
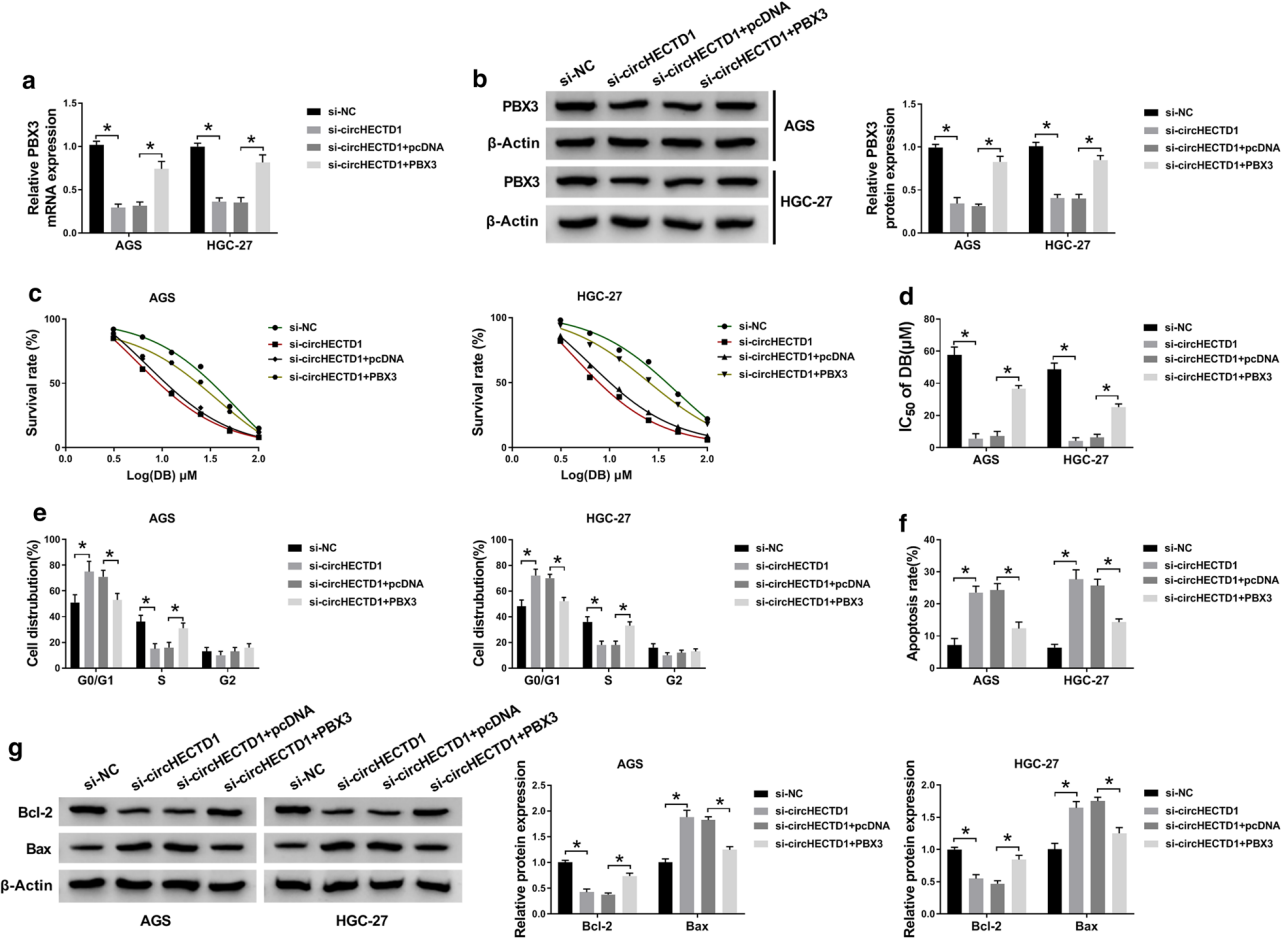
CircHECTD1 mediated the sensitivity of GC cells to DB treatment by regulating PBX3 in GC. **a**–**g** AGS and HGC-27 cells under DB treatment were transfected with si-NC, si-circHECTD1, si-circHECTD1 + pcDNA, or si-circHECTD1 + PBX3, respectively. **a, b** The mRNA and protein levels of PBX3 in transfected GC cells were detected by qRT-PCR and western blot assays. **c**, **d** IC50 values (µM) of transfected cells were determined by MTT assays. **e, f** Flow cytometry assay was conducted to estimate cell cycle distribution and cell apoptosis. **g** The protein levels of Bcl-2 and Bax in cells upon transfection were detected by western blot. All the data from three different experiments were shown as means ± SD. *P < 0.05

The mRNA level of PBX3 was negatively correlated with the expression of circHECTD1 in 25 cases of GC tissues (Fig. 8a). To verify whether circHECTD1 regulated DB-sensitivity of GC cells via regulating miR-137/PBX3 axis, rescue experiments were performed. As displayed in Fig. 8b, c, the mRNA and protein levels of PBX3 were blocked by circHECTD1 knockdown, and such suppression impact could be relieved by miR-137 inhibitor in AGS and HGC-27 cells. Furthermore, PBX3 overexpression partly rescued the decreasing level of PBX3 in GC cells that mediated by si-circHECTD1 (Fig. 9a, b). And the promotion effects of circHECTD1 knockdown on DB-sensitivity, including the decreasing IC50 (Fig. 9c, d), G0/G1 phase arrest (Fig. 9e), and elevated apoptosis cells (Fig. 9f, g), were partly reversed by PBX3 overexpression. Taken together, circHECTD1 acted as an endogenous sponge for miR-137 to regulate the expression of PBX3, thereby suppressing the sensitivity of GC cells to DB treatment.

## Discussion

GC is a serious malignancy in the digestive system, which was a barrier of human health [28]. Until now, the therapeutic approaches for GC, including surgery radiotherapy and chemotherapy, are the major strategies in the clinic [29, 30]. Nevertheless, as the occurrence and application of the chemotherapeutic agents, the efficiency of the traditional strategies were substantially limited in GC [31]. It is urgent to seek novel drugs for cancer treatments. DB has been proved to be connected with human cancer therapy, but low-dose of it was ineffective on cancer cells [5]. Hence, enhancing the sensitivity of DB might provide a new curative approach for GC treatment.

Over the past decades, circRNAs have been regarded as a group of stable and conserved RNAs [32]. A growing body of findings has uncovered the momentous functions of circRNAs, including transcriptional modulation, protein isolation in cancer [33, 34]. Therefore, circRNAs are deemed as the critical mediators in the development of GC by affecting tumor growth and metastasis. Recently, accruing researches have demonstrated the potential use of circRNAs in nervous system disorders and cancers [35, 36]. For example, circHECTD1 served as suggested an oncogene in GC progression by activating glutaminolysis [16]. A previous study also expounded that circRNAs are tightly implicated in drug-resistance in human cancers [37]. Circ_0004015 could mediate drug-resistance by inhibiting miR-1183 in non-small cell lung cancer [14]. In the current research, a marked high level of circHECTD1 was observed in clinical GC samples and cells with respect to corresponding controls, and circHECTD1 silencing curbed the growth of GC tissues *in vivo*, which was agreed with a previous introduction [16]. On this basis, we also further detected the influence of circHECTD1 on DB-sensitivity *in vitro*. Firstly, the absence of circHECTD1 resulted in a significant curb of DB-IC_50_ in AGS and HGC-27 cells. Results from functional assays also presented that circHECTD1 knockdown enhanced DB-sensitivity in GC cells by regulating cell viability, cell cycle distribution, and apoptosis.

Given the function of circHECTD1 in drug-sensitivity, we attempted to explore the targets of it. As is well known, circRNAs served as ceRNAs for miRNAs to absorbing miRNAs [17]. We speculated that circHECTD1 directly targeted certain miRNAs in GC. According to the prediction of starBase, miR-137 was a possible target miRNA of circHECTD1, and subsequent verification tests further determined that circHECTD1 was a sponge of miR-137. In regard to miR-137, it has been implied to act as a tumor suppressor in several cancers, including GC [38, 39]. Consistent with their study, we further discovered that miR-137 was forcefully low-expressed in both GC tissues and cell lines. However, the comprehensive biological functions and drug-sensitive response needed to be identified. Next, we paid attention to the role of miR-137 on DB-sensitivity; the decline of miR-137 could constrain DB-sensitivity in the two GC cells with DB exposure. Apart from that, miR-137 inhibition also eliminated the positive impact of circHECTD1 deletion on DB-sensitivity *in vitro*. Namely, circHECTD1 exerted a negative effect on drug-sensitivity by retarding miR-137 in DB-evoked GC cells.

It is better-known that PBX3 was a cancer-related protein, and it is central to numerous gene molecular networks [40]. A previous study implied that PBX3 strengthened cell metastasis via the MAPK signaling pathway in colorectal cancer [41]. Accordingly, Targetscan software predicted that PBX3 was a candidate gene for miR-137. Also, this assumed relationship between them was verified via dual-luciferase reporter, RIP and RNA pull down assays. Although PBX3 has been identified to be up-regulated in GC [42], the functional role of it in drug-resistance was not explored before. Consistent with previous findings that PBX3 was indeed overexpressed in GC tissues and cells, we further paid attention to discovering the interaction between PBX3 and DB-sensitivity. Functional experiments showed that PBX3 deficiency sensitized DB-treatment in GC cells, and the repressive impact on DB-sensitivity caused by miR-137 inhibitor was partially facilitated by PBX3 deficiency in AGS and HGC-27 cells with DB-stimulation. These data showed that PBX3 was a tumor promoter in GC progression, and it could accelerate cell viability and inhibit cell-cycle arrest and apoptosis in DB-induced GC cells via serving as a target of miR-137. Moreover, the mRNA and protein expression of PBX3 were declined in GC cells after circHECTD1 knockdown, whereas the reduction was regained by miR-137 silence. And overexpression of PBX3 partly reversed the effects of circHECTD1 knockdown-mediated higher sensitivity of GC cells to DB treatment. Taken together, these findings suggested that circHECTD1 could aggrandize DB resistance of GC cells by regulating miR-137/PBX3 axis.

## Conclusions

To sum up, the investigation manifested an unappreciated molecular mechanism of circHECTD1 on DB-sensitivity through adopting miR-137/PBX3 axis via altering cell viability, cell-cycle arrest and apoptosis in DB-induced GC cells, which might provide theoretical support for the application of circHECTD1 as a novel therapeutic strategy for GC patients.

## Data Availability

All data generated or analysed during this study are included in this published article.
